# Supplementary figures and images for: Lenvatinib as First-Line Treatment for Unresectable Hepatocellular Carcinoma: A Systematic Review and Meta-Analysis
Source: Cancers (Basel). 2022 Nov 10;14(22):5525. doi: 10.3390/cancers14225525 (PMC9688932; doi:10.3390/cancers14225525)

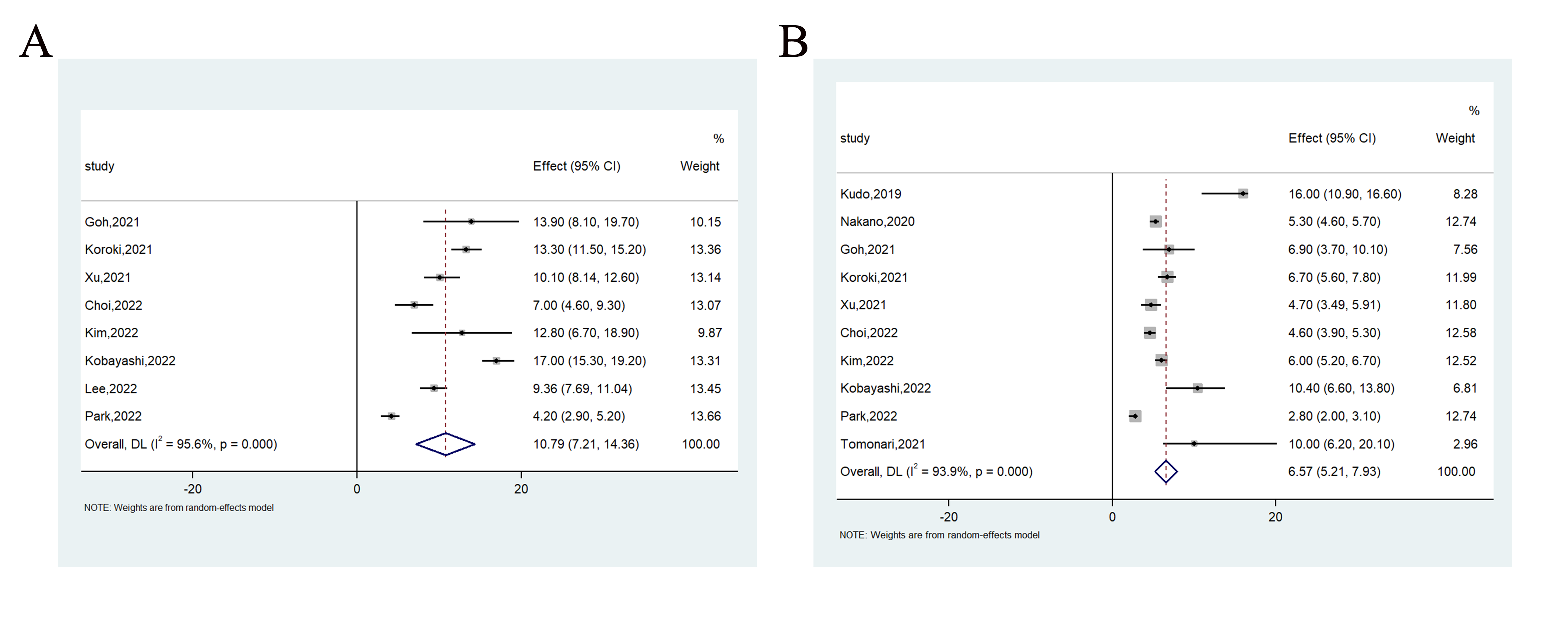

Supplement: Supplementary file 1 [file cancers-14-05525-s001.zip › Supplementary Figure S1.tif]

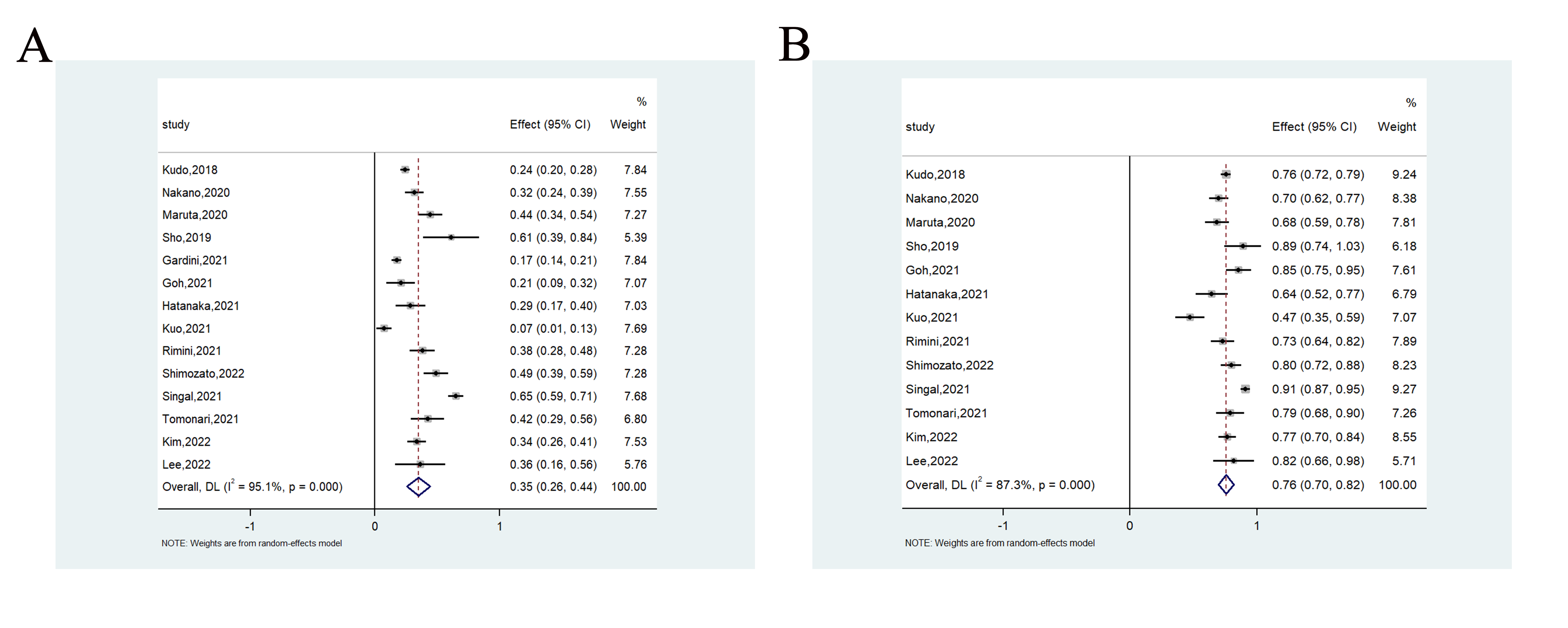

Supplement: Supplementary file 1 [file cancers-14-05525-s001.zip › Supplementary Figure S10.tif]

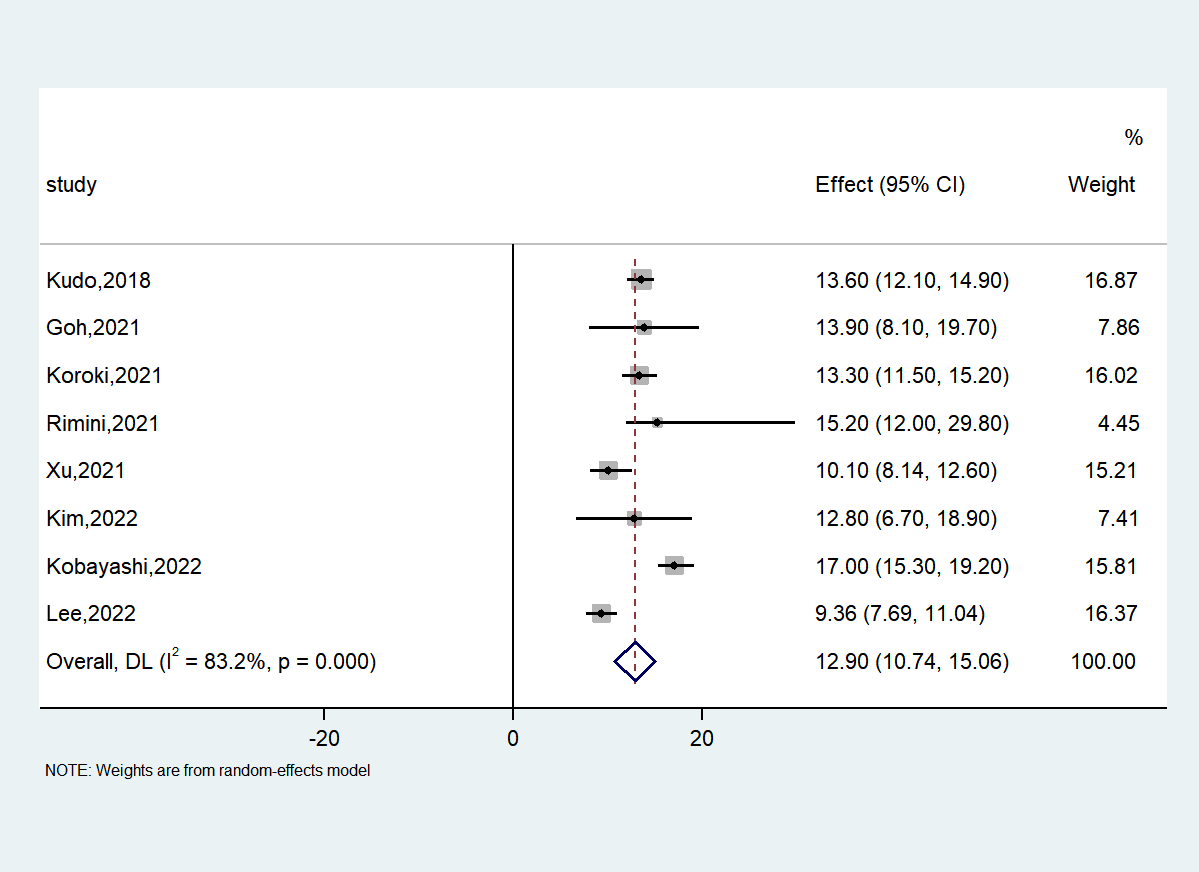

Supplement: Supplementary file 1 [file cancers-14-05525-s001.zip › Supplementary Figure S11.tif]

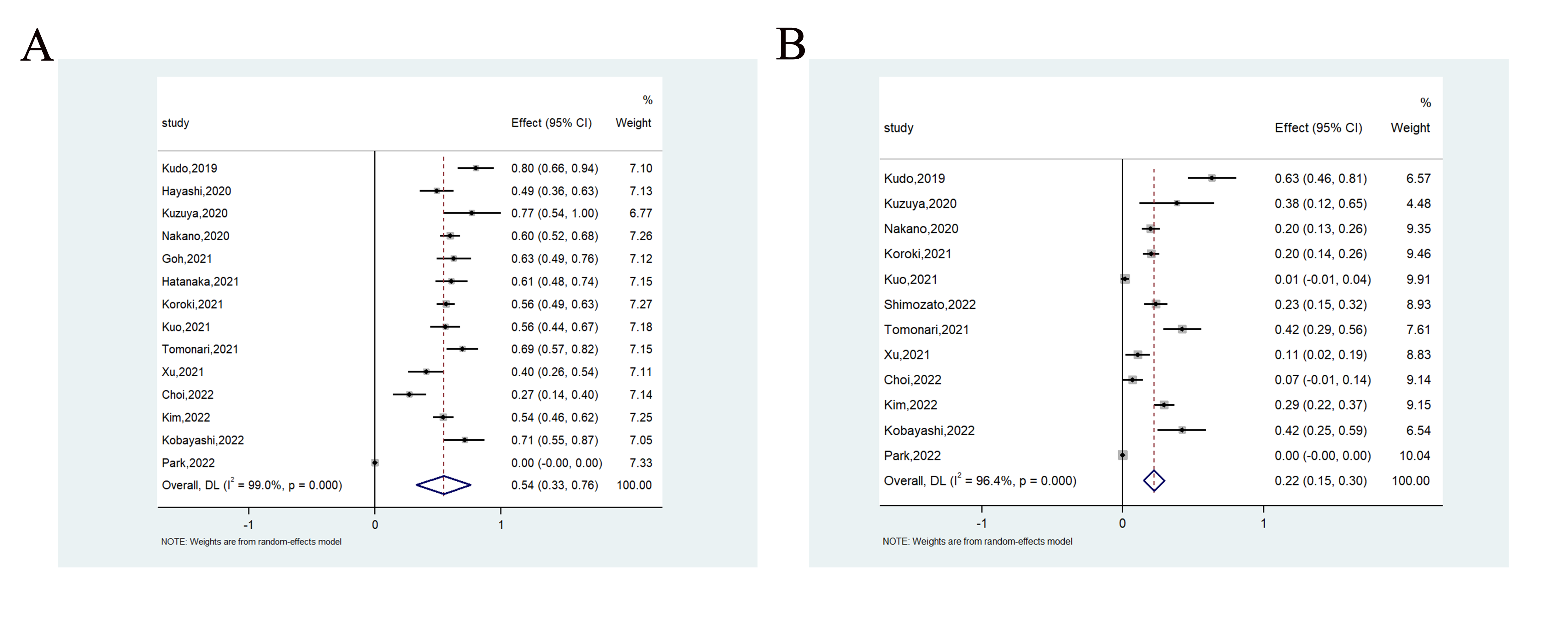

Supplement: Supplementary file 1 [file cancers-14-05525-s001.zip › Supplementary Figure S2.tif]

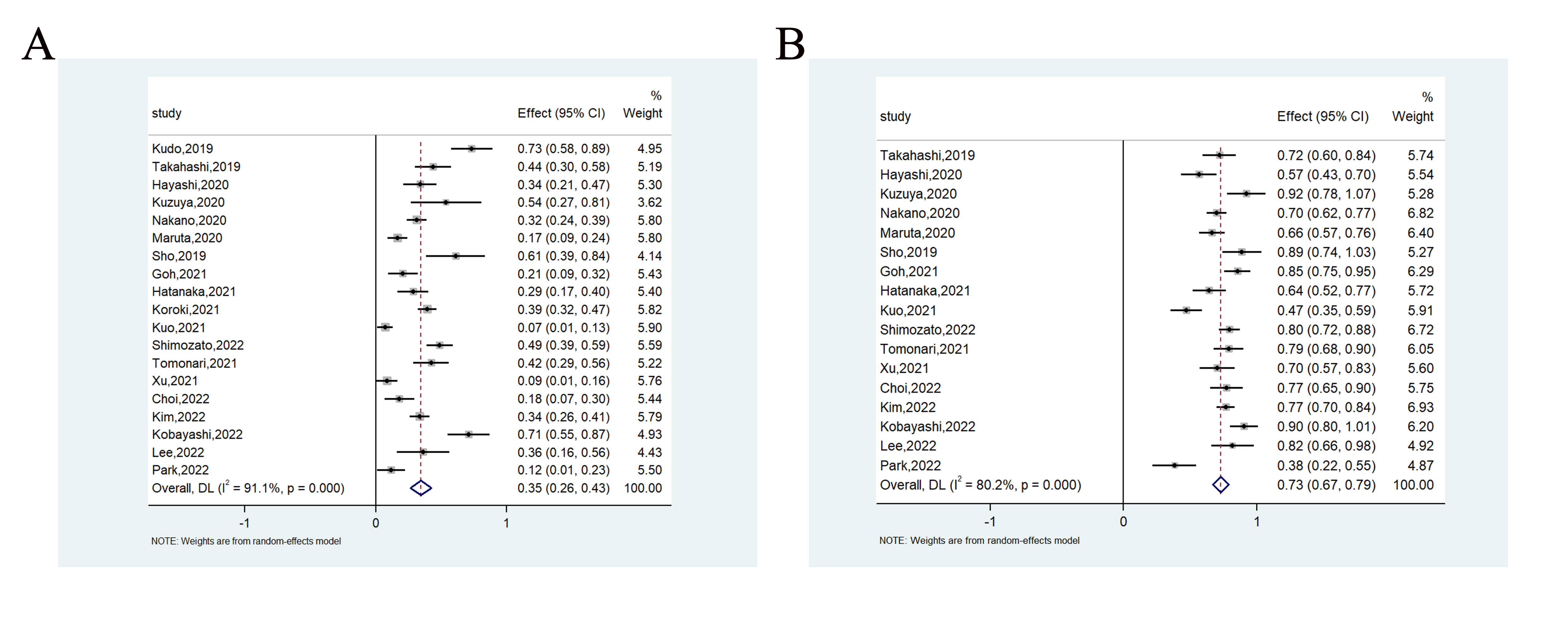

Supplement: Supplementary file 1 [file cancers-14-05525-s001.zip › Supplementary Figure S3.tif]

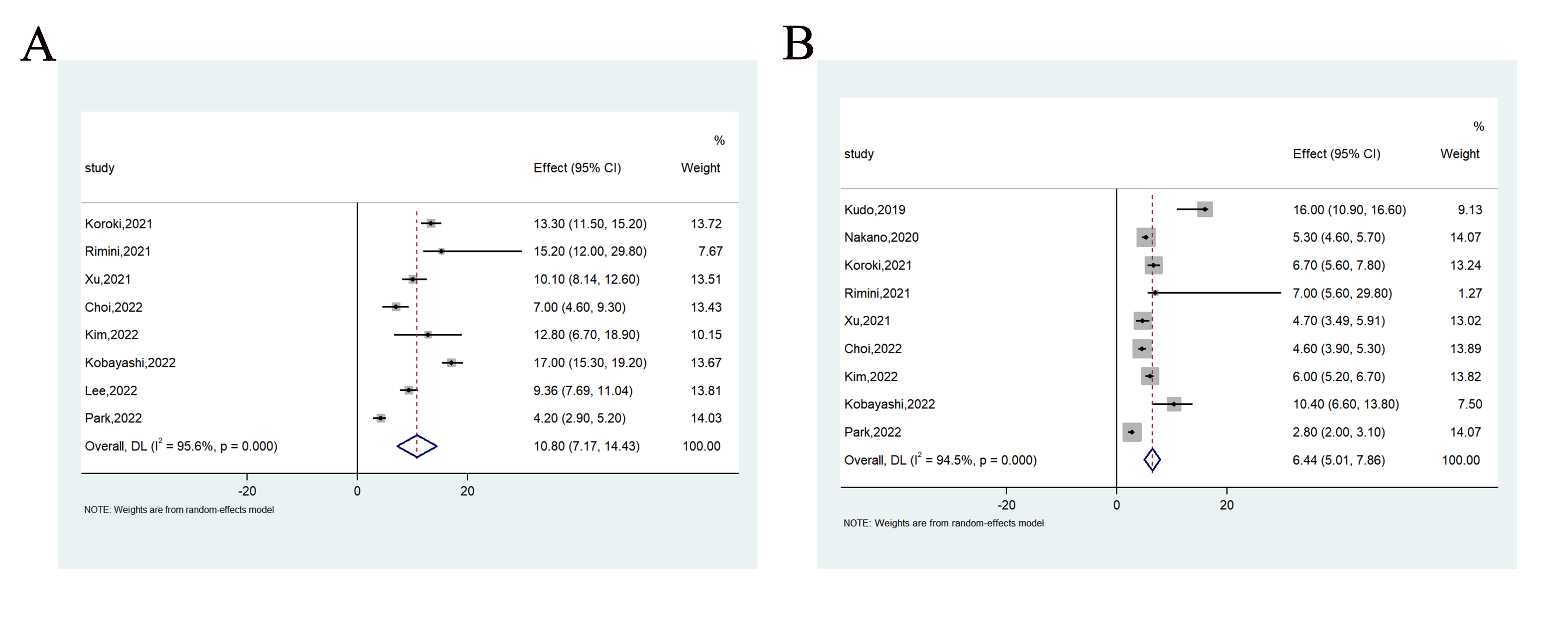

Supplement: Supplementary file 1 [file cancers-14-05525-s001.zip › Supplementary Figure S5.tif]

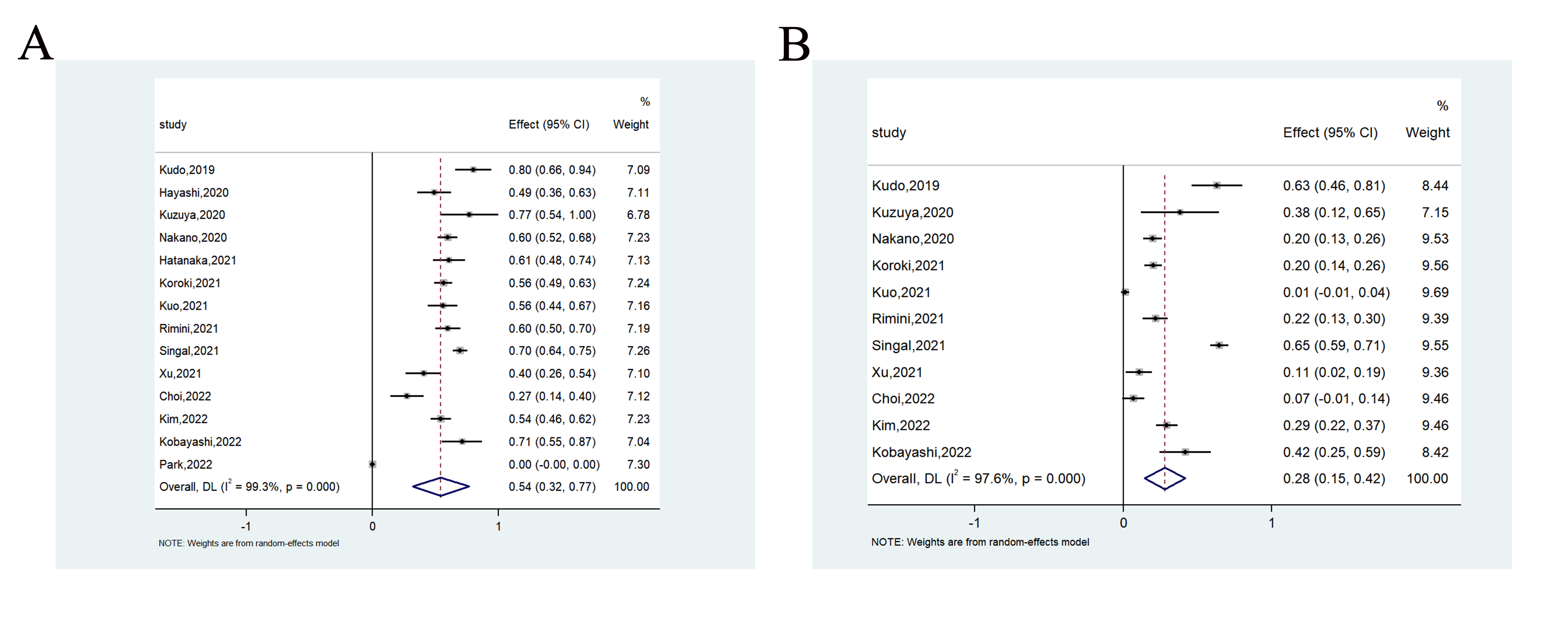

Supplement: Supplementary file 1 [file cancers-14-05525-s001.zip › Supplementary Figure S6.tif]

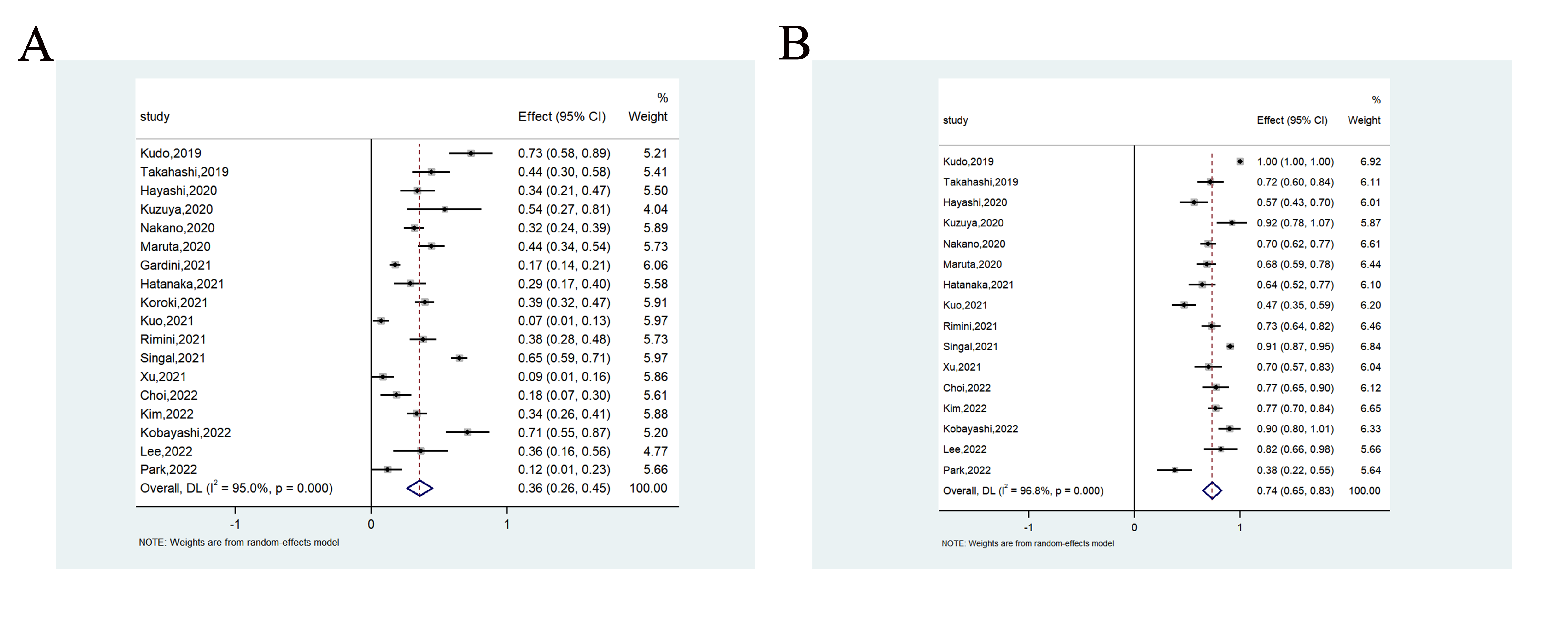

Supplement: Supplementary file 1 [file cancers-14-05525-s001.zip › Supplementary Figure S7.tif]

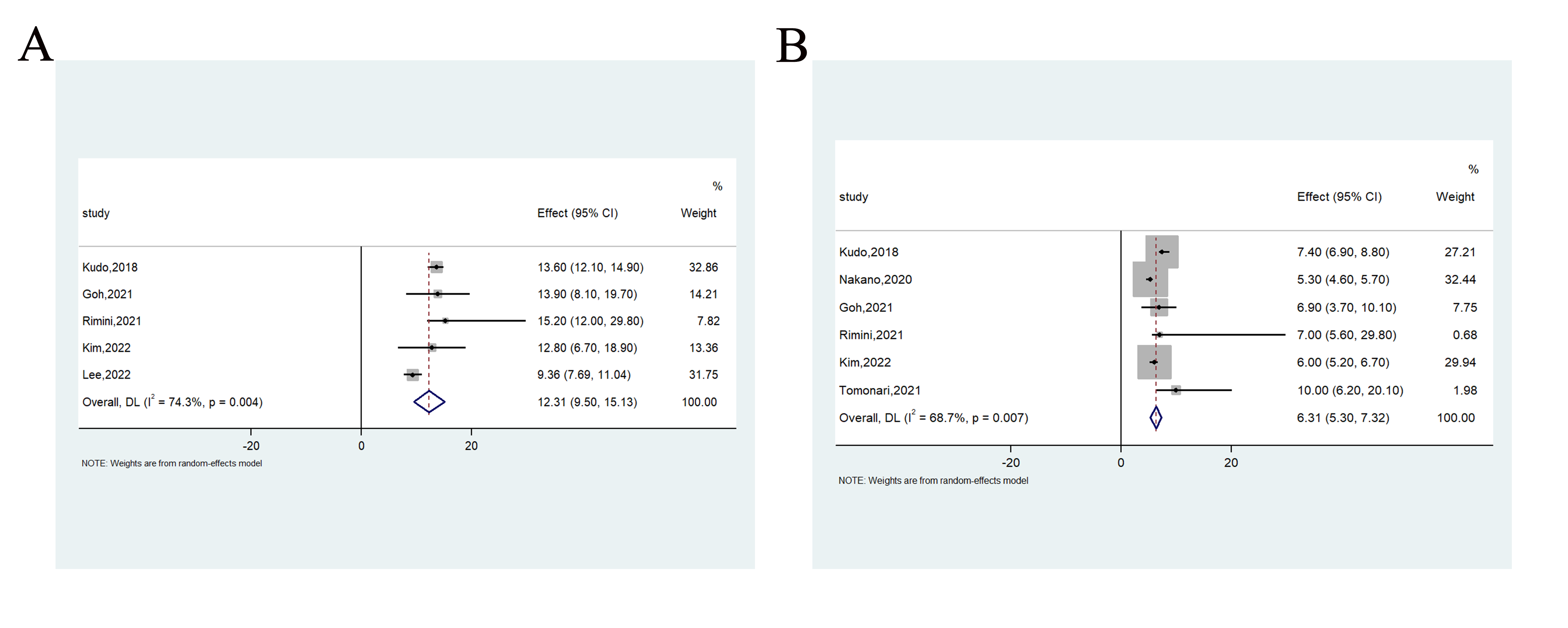

Supplement: Supplementary file 1 [file cancers-14-05525-s001.zip › Supplementary Figure S8.tif]

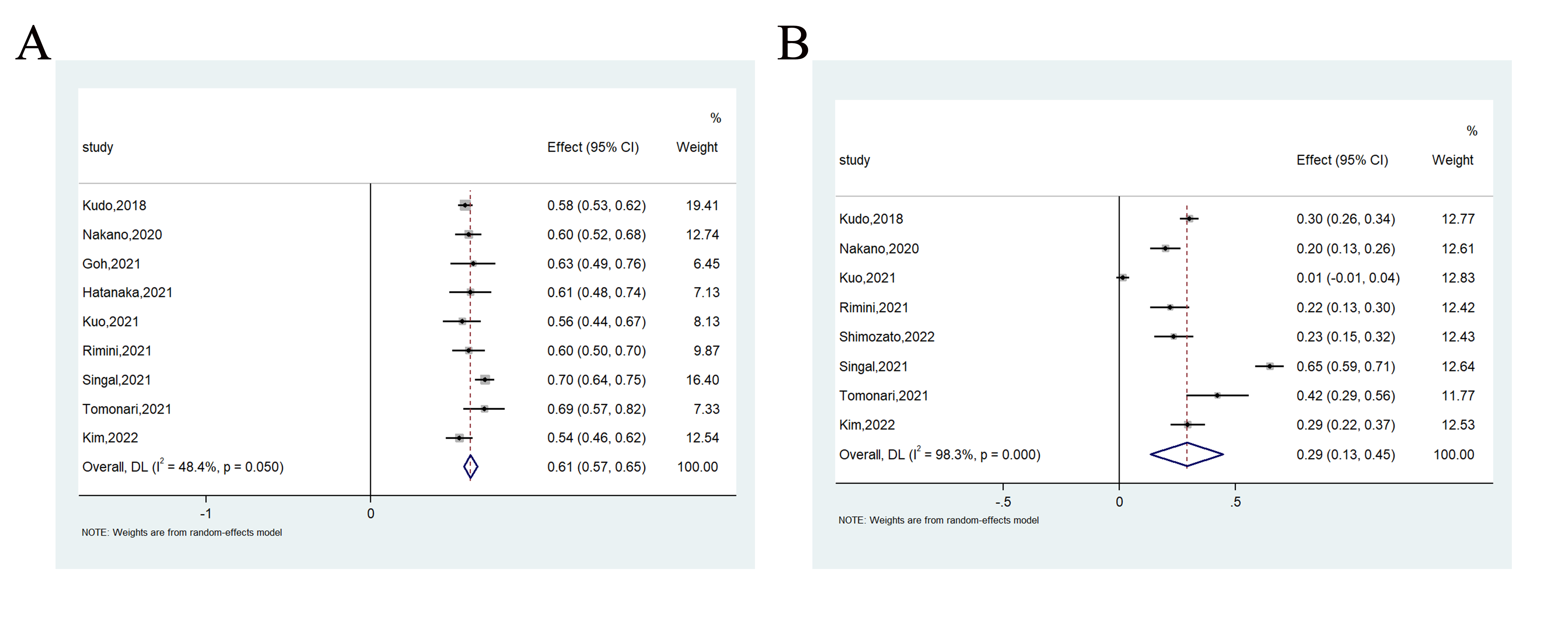

Supplement: Supplementary file 1 [file cancers-14-05525-s001.zip › Supplementary Figure S9.tif]
